# Optimization of TiC Content during Fabrication and Mechanical Properties of Ni-Ti-Al/TiC Composites Using Mixture Designs

**DOI:** 10.3390/ma11071133

**Published:** 2018-07-04

**Authors:** Dong-Jin Lee, Jae-Ha Park, Myung-Chang Kang

**Affiliations:** 1 Graduate School of Convergence Science, Pusan National University, Busan 46241, Korea; eastjin218@naver.com; 2 Software Division South Office, IREATECT Co., Busan 49465, Korea; jhpark@minitab.co.kr

**Keywords:** Ni-Ti-Al/TiC, tribology behavior, spark plasma sintering, mixture designs, TiC content optimization

## Abstract

Ni-Ti-Al alloys are highly promising materials for use in high-temperature structural materials. However, minimal research has been conducted to improve the associated mechanical properties through secondary phase addition. In this study, Ni-Ti-Al/TiC composites were fabricated at a pressure of 40 MPa and a sintering temperature of 1050 °C using spark plasma sintering. The microstructure and interfacial structure were analyzed by scanning electron microscopy, energy dispersive X-ray spectroscopy, and X-ray diffraction analysis. Microscopic analysis revealed that TiC particles interacted with Ti and Al, resulting in the formation of Ti_2_AlC, which promoted chemical metallurgical bonding between the Ni–Ti–Al alloy and TiC. Wear characteristics were measured using the wear test with a ball on disk. It was confirmed that the 40 wt % specimen had the highest hardness due to pores generated inside, but the wear amount was relatively high. The mixture design of a minitap was proceeded using hardness, bending strength, and wear loss. An optimum composition ratio of 32.16 wt % was determined using the composite desirability of the three properties.

## 1. Introduction

The potential application of Ni-Ti-Al-based alloys for use in aerospace and high-temperature structural materials is acknowledged with respect to their low density, high strength at high temperatures, and high corrosion resistance. Initial studies improved the strength of Ni-Ti-based shape memory alloys at high temperatures by preparing Ni-Ti-Al by the substitution of a small amount of Ti with Al [1,2]. Furthermore, a recent study was conducted to improve the corrosion resistance at high temperatures and prevent a deterioration in mechanical properties, in which less than 10 at% Al was used for the precipitation of the Ni_2_TiAl phase and NiTi phase [3].

To improve the mechanical properties of Ni-Ti-Al-based alloys, metal elements, such as Mo, Nb, Hf, Zr, B, and Re, have been added to alloys [4,5,6], and these properties have also been evaluated at room temperature. For example, the refractory alloy, Mo, was added at the interface between TiAl and NiAl to improve yield strength and compression strength, and Nb was added to improve the oxidation resistance. To improve the mechanical properties at high temperature, Ni-45Ti-5Al-2Nb-1Mo alloy was prepared by adding a small amount of at% [7]. In addition, studies have been conducted to improve the compressive strength at room temperature by preparing (Ti, Al) 2 Ni and (Zr, Ti, Al) 2 Ni solid phase by adding 8 at% of Zr [8], and numerous studies have investigated methods to improve high-temperature properties [3,9,10].

However, it is necessary to research the application of metal- or ceramics-based composites in extreme environments due to the development of advanced industries. Mechanical properties research has previously been limited to compressive strength tests (because these composites are high-temperature structural materials), but limited research has used room temperature to investigate the wear loss of the product or evaluate its mechanical properties. Furthermore, mechanical properties have been evaluated under limited conditions, due to the uniform composition ratio of the added materials; however, few studies have aimed to find the optimal composition ratio because composition ratio tests are expensive and time-consuming.

Therefore, in this study, TiC [11,12] with various composition ratios is added to Ni-Ti-Al and sintered using identical sintering conditions in all experiments. The material properties are analyzed by measuring the microstructure, composition distribution, phase, and density; and mechanical properties, such as bending strength, hardness, and wear loss are measured and applied to the mixture design. The optimum content of TiC is obtained using the composite desirability of the mixture design.

## 2. Materials and Methods

In this experiment, Ti (99.96%, 45 μm), Al (99.95%, 45 μm), Ni (99.9, 45 μm), and TiC (99.95%, 3 μm) were employed at the composite ratios shown in Table 1. In the chemical reaction presented in Equation (1), all of the TiC can disappear during sintering, so that the conditions were excepted the composition of TiC 10 wt %. SUS balls with diameters of 10 mm were mixed at a ball/powder ratio (BPR) of 10:1 for 10 h.

(1) $$\mathrm{Ti}+Al+\mathrm{TiC}\;\to {\;\mathrm{Ti}}_{2}\mathrm{AlC},$$

The Ni-Ti-Al/TiC powders were charged into a graphite mold with a diameter of 30 mm, pre-formed by hand press, and then sintering using spark plasma sintering (SPS: DR. SINTER SPS-825, FUJI-SPS, Gothenburg, Japan). In the preliminary experiment, the sintered material exited from the graphite mold at a pressure of 40 MPa and at temperatures of 1100 °C or higher and adhered to the disk. Therefore, sintering was subsequently conducted by maintaining the pressure at 40 MPa, the temperature elevation at 100 °C/min, and the sintering temperature of 1050 °C for 10 min in a vacuum atmosphere. The densities of the specimens were measured using the Archimedes method, and the average of five measured values was used to calculate the theoretical density. Relative density values were calculated using Equation (2) to determine the porosity of specimens,

(2) $$K=\frac{\rho }{\rho_{0}}\times 100\%,$$

where *K* is the relative density, ρ is measured density, and $\rho_{0}$ is theoretical density. X-ray diffraction (XRD: D8-ADVANCE, Bruker, MA, USA) was used to analyze the phase as the content of TiC increased. The wear amount of the specimen was measured by a ball-on-a-disk-type wear tester (High temperature wear tester, R&BCo. Ltd., Daejeon, Korea); the microstructure and indentation of the wear specimen were measured using scanning electron microscopy (SEM, S-4800, HITACHI, Chiyoda, Japan) and energy dispersive X-ray spectroscopy (EDS: EDAX, Bruker, MA, USA); hardness was measured using a Vickers hardness tester (VMT-X7, Matsuzawa, Akita, Japan); and bending strength was measured using the indentation fracture method at a load of 5N. Test specimens were prepared in 4mm × 8mm × 24 mm according to the KS D ISO 3325 standard [13] using a discharge wire. They were then measured three times, and the bending strength was expressed using the average, maximum, and minimum range. 

**Figure 1:**
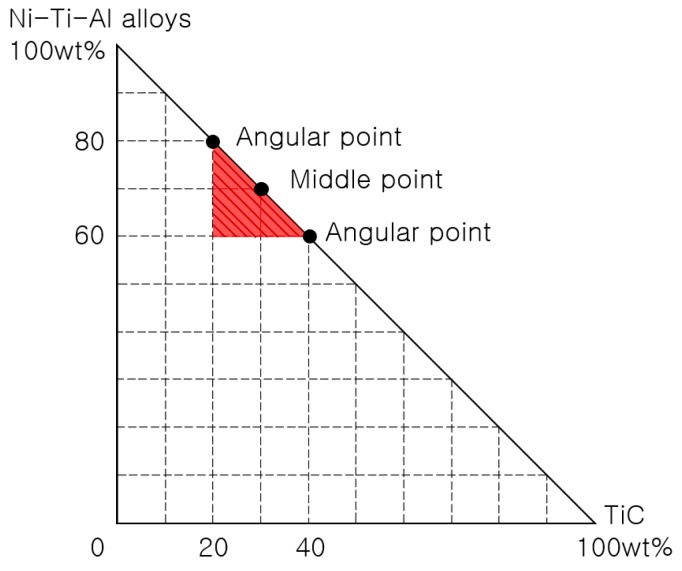
Schematic diagram of Ni-Ti-Al/TiC composite ratios in mixture design using Minitab 17.

**Figure 2:**
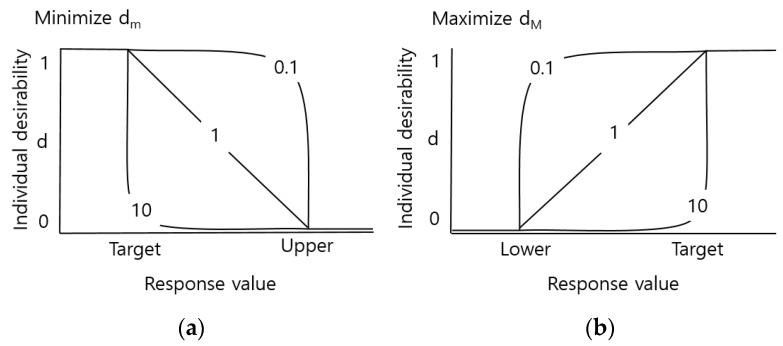
Principle schematic diagram of applying weight of desirability function in mixture design. (**a**) Minimize d_m_; (**b**) Maximize d_M_.

The mixture design was processed in Minitab 17 (Minitab Inc., State College, PA, USA) using the measured bending strength, hardness, and wear loss. The vertex design represents the mixture design when the composition ratio is limited to the maximum and minimum, and Figure 1 shows a composite rate schematic diagram of the Ni-Ti-Al/TiC composite vertex design at ratios of 80:20, 60:40 wt % (angle point), and 70:30 wt % (middle point). As the design utilizes two deviations and variances, it was performed twice for each condition. The middle point was added to confirm the tendency between the two points and proceeded six times in total. The degree of similarity between the model and actual data were analyzed using the measured values of hardness, bending strength, and wear loss. The maximum (d_M_) was selected by setting the minimum value ($L_{i}$) and target value ($T_{i}$) of hardness and bending strength, and the minimum (d_m_) was selected by setting the maximum value ($H_{i}$), and target value of wear loss. The composite desirability is expressed as a combination of the maximum d or minimum d of individual desirability (d). The desirability function weight $\left(r_{i}\right)$ was calculated for individual desirability (d) and composite desirability when emphasizing the importance of each expected response value ($y_{i}$), and the function is shown in Figure 2. A range between 0.1 and 10 is possible; if the value is larger than 1 then the reaction value is closer to the target value, whereas if it is lower than 1 then the optimum condition is found (even if the reaction value does not move close to the target value).

## 3. Results and Discussion

### 3.1. Optimization of TiC Contents through the Mixture Design

The reaction optimization tool can identify a single response or a combination of input variables and provide optimization of individual desirability and composite desirability. Individual desirability (d) is calculated in the range from 0 to 1 (where 1 is ideal and maximum (d_M_) and minimum (d_m_) values are calculated based on formula (3). d is the distance d between maximum value ($H_{i}$), minimum value ($L_{i}$), the target value ($T_{i}$) and the expected response value ($y_{i}$). The composite desirability (D) is calculated in the range from 0 to 1 using Equation (4), by summing each response satisfaction, d, shown in Equation (3),

(3) $${\mathrm{maximize}\;d}_{M}={\left(\frac{\left(y_{i}{-L}_{i}\right)}{{(T}_{i}{-L}_{i})}\right)}^{r_{i}}{,\;\mathrm{minimize}\;d}_{m}={\left(\frac{\left(H_{i}{-y}_{i}\right)}{{(H}_{i}{-T}_{i})}\right)}^{r_{i}},$$

(4) $$\mathrm{composite}\;\mathrm{desirability}\;D={\left(d^{r_{1}}{}_{1}{\times d}^{r_{2}}{}_{2}\times \ldots \times d_{n}\right)}^{1/W},$$

where $r_{i}$ is the weight of the desirability function of the ith response; and W is Σ$r_{i}$.

**Figure 3:**
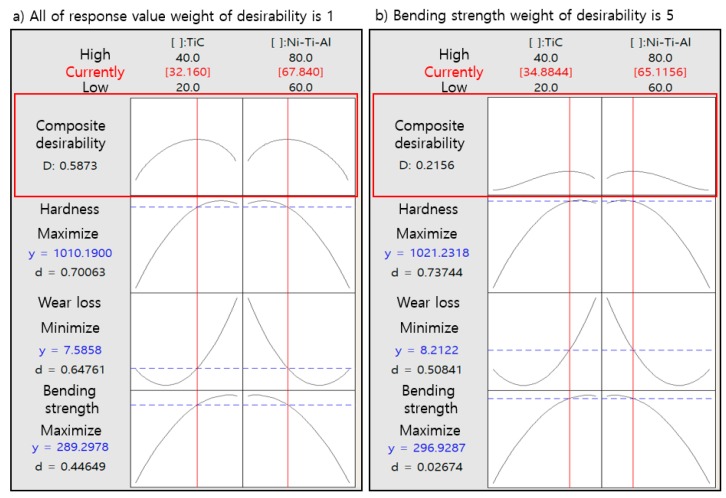
Response optimizer for mixture design using Minitab 17. (**a**) All of response value weight of desirability is 1; (**b**) Bending strength weigh of desirability is 5.

Figure 3 shows the optimization of the composition using the reaction optimization tool. This experiment was conducted by choosing the same weight as 1 to determine the characteristics of mechanical properties, but not with respect to their commercialization. The optimum composition was selected as 0.5873 (Ni-Ti-Al: 67.84 wt %, TiC: 32.16 wt %). However, individual satisfaction with respect to bending strength was low because of the large difference between the target and minimum values. Nevertheless, this composition was selected because of the slight increase in individual satisfaction with respect to wear loss and hardness compared to the increase in individual satisfaction with respect to bending strength. This experiment was used to confirm the feasibility of characterization and optimization according to the composition of TiC, and it is acknowledged that the optimal composition ratio may vary depending on the weight. In Figure 3b, the bending-strength weight of the desirability function is selected as 5.

### 3.2. Effect of TiC Contents on Density and XRD Pattern Microstructure of Ni-Ti-Al/TiC Composites

Table 2 shows the theoretical, measured, and relative densities of the Ni-Ti-Al/TiC composites. As the TiC content increased, the theoretical and measured density values decreased with respect to the decrease in the composition ratio of Ni, which has a relatively high density. In addition, sintering under the same sintering conditions shows that the relative density decreased under the condition of 40 wt % TiC. This is attributed to an insufficient penetration of the low contact angle of Ni-Ti-Al between the particles of TiC, and the formation of pores during sintering due to the increase in TiC.

**Figure 4:**
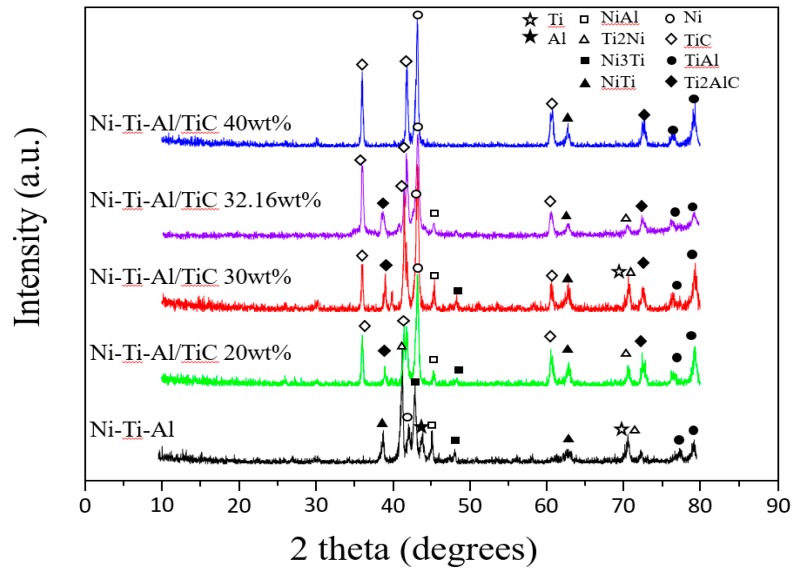
XRD analyses of Ni-Ti-Al/TiC composition and Ni-Ti-Al alloy.

Figure 4 shows the phase composition of the mixes and sintered composites. The main phases found in the mixes are Ni, Ti, Al, and TiC, and phases found in the sintered composites are NiTi, Ti_2_Ni, TiC, Ni_3_Ti, TiAl, Ti_2_AlC complex phases and Ti, Ni, Al phases. As the content of TiC increased under the same sintering condition, the peak of TiC increased in the range of 20 wt % to 40 wt % but the Ti_2_AlC peak decreased at 40 wt %. It is assumed that the TiC powder was incompletely sintered at the interface of Ni-Ti-Al and TiC in these reactions [12]. XRD showed that at TiC peak values of 20 wt % and 30 wt %, TiC was synthesized to Ti_2_AlC. In addition, with increased amounts of TiC, values of Ni_3_Ti and NiTi decreased and the Ni peak rose due to the decreased Ni reaction.

**Figure 5:**
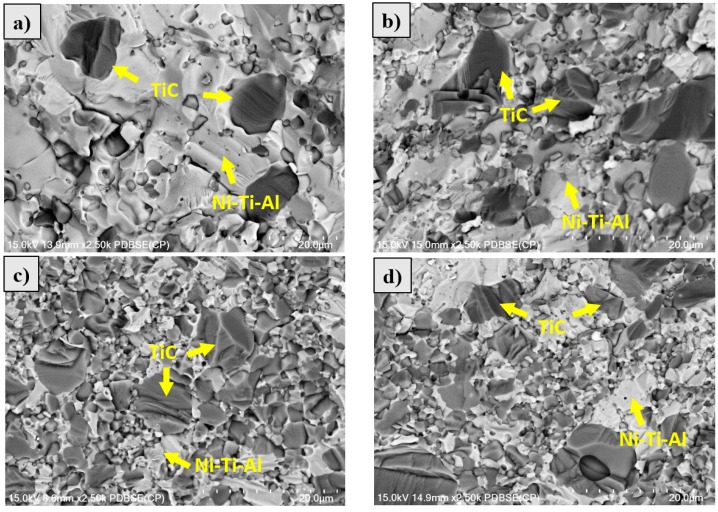
Microstructure of Ni-Ti-Al/TiC composites by SPS BSE of: (**a**) TiC content 20 wt %; (**b**) TiC content 30 wt %; (**c**) TiC content 32.16 wt %; (**d**) TiC content 40 wt %.

Figure 5 shows the microstructure of the fabricated Ni-Ti-Al/TiC composites with TiC contents of 20, 30, 32.16, and 40 wt %. The TiC particle was uniformly distributed in the Ni-Ti-Al alloy matrix at TiC contents of 20 and 30 wt %. The number of fine-sized (3–5 µm) TiC particles increased abruptly at 32.16 wt % and 40 wt %. However, TiC and Ni-Ti-Al were not uniformly distributed at 40 wt % and were found to be united. Table 2 and Figure 5 show that Ni-Ti-Al did not penetrate sufficiently between the TiC particles as the content increased, resulting in pores in the interface of TiC and Ni-Ti-Al.

**Figure 6:**
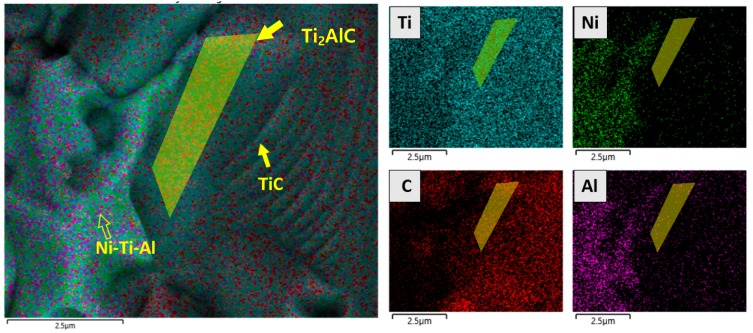
SEM images and EDS elemental mapping analysis of Ni-Ti-Al/TiC composites.

**Figure 7:**
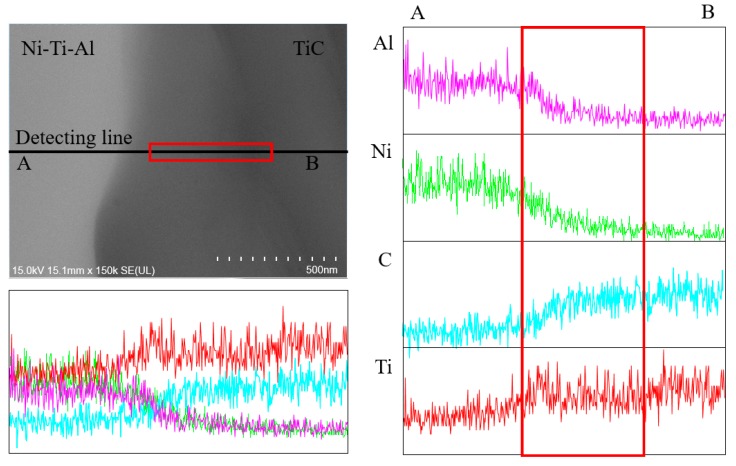
Microstructure and EDS analysis of interface between TiC and Ni-Ti-Al alloy.

Figure 6 shows the microstructure and distribution of elements across the interface between the TiC and the Ni–Ti–C alloy, as determined by EDS. EDS mapping analyses revealed a distribution gradient for the elemental composition: Ti was distributed as a whole, and Ni, Al, and C were partially concentrated. The distributions and phases of TiC and Ni-Ti-Al alloys were confirmed and are shown in Figure 4 and Figure 6. The formation of Ti2AlC was observed using the compositional distribution at the interfacial bonding between the Ni-Ti-Al and TiC. Results showed an almost complete lack of Ni in the interface between Ni-Ti-Al and TiC but a region where Al and C were common, which is considered to be a Ti2AlC layer with a 0.5 to 1 μm interval. Figure 7 shows a line scan analysis using EDS. Due to the nature of high-energy sintering equipment, SPS, the components are seen to be diffused and were measured as a whole, but it was possible to obtain high peaks at key locations. The decrease in the Ni peak in the interface region of Ni-Ti-Al and TiC was larger than the decrease in the Al peak. In addition, the gray section was confirmed to be a Ti2AlC layer of 0.5 μm, due to the distribution peak of C.

(5) $$3\mathrm{Ni}+\mathrm{Ti}\;\to {\mathrm{Ni}}_{3}\mathrm{Ti},$$

(6) $$3\mathrm{Ti}+2\mathrm{Al}+2\mathrm{Ni}\to \;\mathrm{TiAl},\;\mathrm{NiAl},\;{\mathrm{Ti}}_{2}\mathrm{Ni},$$

### 3.3. Mechanical Properties of Ni-Ti-Al/TiC Composites

**Figure 8:**
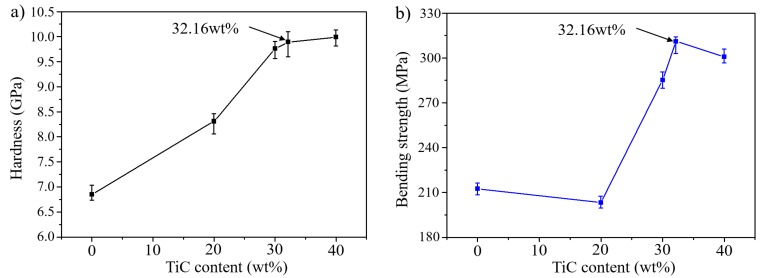
Effect of TiC contents on: (**a**) macro hardness and (**b**) bending stress.

Figure 8a shows a graph of changes in hardness with increases in the TiC composition. Hardness measured using a Vickers hardness meter was 8.3 GPa at 20 wt % TiC, but it increased to 9.75 at 30 wt %, and a further slight increase was detected at 40 wt % (from 9.75 GPa to 9.98 GPa). Figure 2 and Figure 3 show that the TiC distribution was higher on the surface, and higher hardness was maintained with a content of 40 wt % due to the higher distribution of TiC on the surface; however the Ni-Ti-Al alloy was not infiltrated into the TiC particle and the surface was therefore easily broken due to the occurrence of the pores and an inactive interface.

Figure 8b shows the bending strength according to the composition ratio, and it is evident that the value of the bending strength increases with an increasing TiC content. However, the strength at TiC 20 wt % is lower than that of the conventional Ni-Ti-Al alloy. The bending strength was increased in relation to the formation of Ti_2_AlC, and the formation of the NiTi phase was inhibited with an increase in the number of TiC particles. This reason was presumed bending strength of the base metal and 30 wt %. The interfacial bonding energy increased for the TiC and Ni-Ti-Al alloy during the Ti_2_AlC phase. Figure 2 shows that when the content of TiC increased, formation of the NiTi phase was inhibited, and Ti_2_AlC was produced. However, the bending strength at 20 wt % and the bending strength of the base metal overlapped in the maximum superficial value range, which suggests that the formation of NiTi was suppressed but the formation of Ti_2_AlC was not smooth. The bending strength increased with an increase in the TiC content: the highest bending strength was 311 MPa at the optimization composite ratio of 32.16 wt %. However, although the hardness value is high, the bending strength decreased due to cracks relating to internal pores [14].

**Figure 9:**
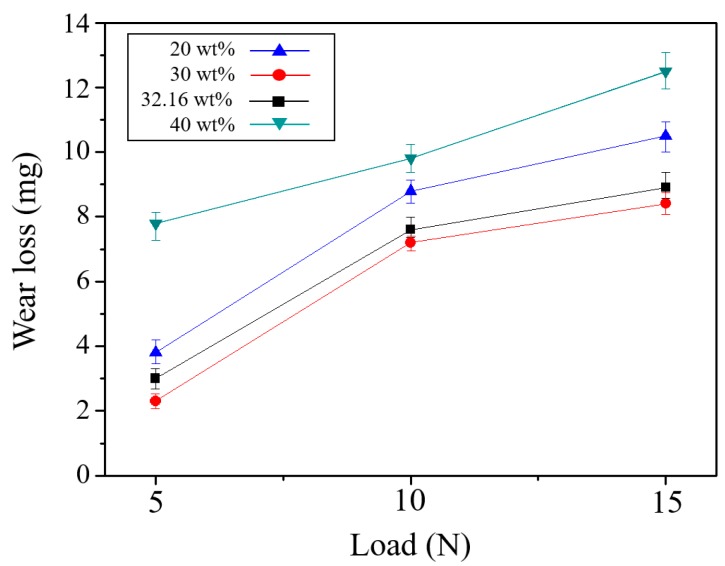
Effect of TiC content on wear loss according to load various.

Figure 9 shows the amount of wear at each TiC composition ratio according to a change in load in the ball-on-disk type abrasion test: the amount of wear increased with an increase in the load. The amount of abrasion was lowest at a TiC content of 30 wt % and highest at 40 wt %. There was a decrease in the abrasion amount at 30 wt % compared to 20 wt % due to the increase in hardness and bending strength. Although the TiC specimen at 40 wt % had a high density due to use of the high plasma density sintering method, the Ni-Ti-Al base material could not sufficiently bond with the TiC. Therefore, the abrasion rate also increased rapidly when the TiC particles dropped out after the load increased [14,15]. In addition, the optimum condition of 32.16 wt % caused more abrasion than at 30 wt %, but the difference was only slight.

**Figure 10:**
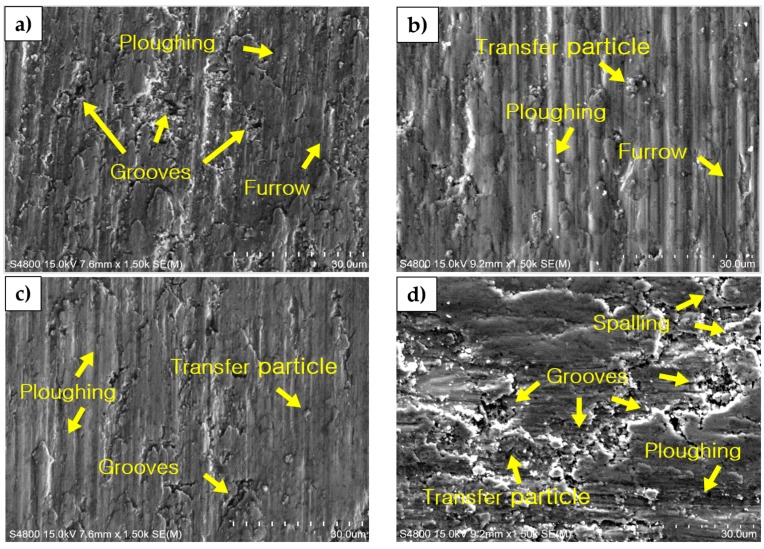

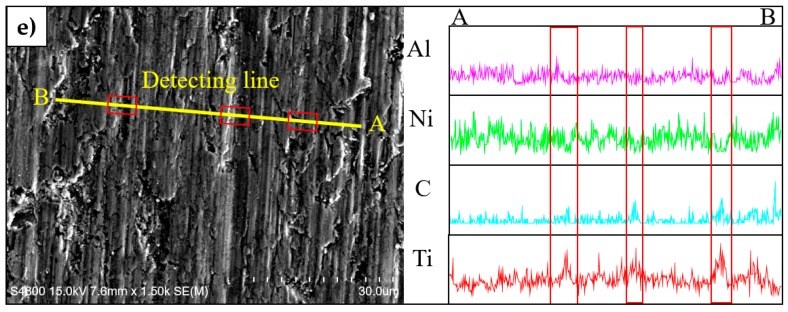
Abrasion surface of Ni-Ti-Al/TiC composites by SPS SEM image of (**a**) TiC content of 20 wt %; (**b**) TiC content of 30 wt %; (**c**) TiC content of 32.16 wt %; (**d**) TiC content of 40 wt %; and EDS analysis showing (**e**) TiC content of 30 wt %.

Figure 10 shows photographs of the surfaces of wear test specimens. The cracks and traces of the wear tracks are more highly evident at 20 wt % and 40 wt % than at 30 wt %, and craters are evident at 40 wt %. It was confirmed that the unsintered TiC particles were separated from each other and that secondary wear had occurred. In addition, the result of EDS shown in Figure 10e evidences the reduction in areas of abrasion at 30 wt %, which relates to the high TiC composition [15,16]. 

## 4. Conclusions

TiC was added to an Ni-Ti-Al alloy, which is a high-temperature structural material, and hardness, bending strength, and wear loss were measured at room temperature. The optimum condition was determined using the mixture synthesis method. The optimum composition ratio is considered to be a factor that can be used to improve the durability life of the product. The composition using the highest composite desirability was selected for all three compositions with high individual desirabilities, which thus suggests the possibility of optimization.

1. The Ti_2_AlC phase was increased with the addition of TiC, but the Ti_2_AlC phase was not formed sufficiently between the interfaces due to the failure of TiC particles to penetrate smoothly at 40 wt %. High densification was achieved using the spark plasma sintering method. However, density was reduced with an increase in the TiC content, and pores were generated at interfaces between TiC and Ni-Ti-Al.
2. The hardness value increased with an increase in the TiC content, and the highest hardness value was measured at 40 wt %. In addition, TiC was saturated on the surface at 40 wt % and an increase range of hardness was decreased from 9.75 GPa to 9.98 GPa.
3. The bending strength was increased due to the formation of Ti_2_AlC, and the formation of the NiTi phase was inhibited with an increase in the TiC particles. The interfacing bonding energy increased between the TiC and Ni-Ti-Al alloy during the Ti_2_AlC phase. The Ti_2_AlC peak at 32.16 wt % was superior to that of the other content materials and the bending strength was superior at 311 MPa.
4. The TiC content has a significant effect on the wear resistance of the composites. At a TiC content of 30 wt %, the weight loss of the composites reached a minimum value of 2.2 mg. Secondary wear occurred with the TiC particle by spalling at TiC 40 wt %.

## Figures and Tables

**Table 1:** Chemical composition of mixed powders (wt %).

Sample	Ni	Ti	Al	TiC
Ni-Ti-Al/20 wt % TiC	40	36.8	3.2	20
Ni-Ti-Al/30 wt % TiC	35	32.2	2.8	30
Ni-Ti-Al/40 wt % TiC	30	27.6	2.4	40

**Table 2:** Density of Ni-Ti-Al/TiC composition.

Sample	Theoretical Density (g/cm^3^)	Measured Density (g/cm^3^)	Relative Density (g/cm^3^)
Ni-Ti-Al/20 wt % TiC	5.59	5.52	98.8%
Ni-Ti-Al/30 wt % TiC	5.50	5.41	98.4%
Ni-Ti-Al/40 wt % TiC	5.41	5.25	97.1%
Ni-Ti-Al/32.16 wt % TiC	5.48	5.37	98.1%
